# The Grapevine Uncharacterized Intrinsic Protein 1 (VvXIP1) Is Regulated by Drought Stress and Transports Glycerol, Hydrogen Peroxide, Heavy Metals but Not Water

**DOI:** 10.1371/journal.pone.0160976

**Published:** 2016-08-09

**Authors:** Henrique Noronha, Diogo Araújo, Carlos Conde, Ana P. Martins, Graça Soveral, François Chaumont, Serge Delrot, Hernâni Gerós

**Affiliations:** 1 Centro de Investigação e de Tecnologias Agro-ambientais e Biológicas CITAB, Vila Real, Portugal; 2 Centre of Molecular and Environmental Biology (CBMA), Department of Biology, University of Minho, Braga, Portugal; 3 i3S - Instituto de Investigação e Inovação em Saúde, Universidade do Porto, Porto, Portugal; 4 IBMC; Instituto de Biologia Molecular e Celular, Universidade do Porto, Porto, Portugal; 5 Research Institute for Medicines and Pharmaceutical Sciences (iMed.UL) University of Lisbon, Lisbon, Portugal; 6 Institut des Science de la Vie, Université Catholique de Louvain, Louvain-la-Neuve, Belgium; 7 INRA, ISVV, Ecophysiologie et Génomique Fonctionnelle de la Vigne, UMR 1287, Université de Bordeaux, Villenave D’Ornon, France; University of Vigo, SPAIN

## Abstract

A MIP (Major Intrinsic Protein) subfamily called Uncharacterized Intrinsic Proteins (XIP) was recently described in several fungi and eudicot plants. In this work, we cloned a XIP from grapevine, *VvXIP1*, and agrobacterium-mediated transformation studies in *Nicotiana benthamiana* revealed that the encoded aquaporin shows a preferential localization at the endoplasmic reticulum membrane. Stopped-flow spectrometry in vesicles from the *aqy*-null yeast strain YSH1172 overexpressing *VvXIP1* showed that VvXIP1 is unable to transport water but is permeable to glycerol. Functional studies with the ROS sensitive probe CM-H_2_DCFDA in intact transformed yeasts showed that VvXIP1 is also able to permeate hydrogen peroxide (H_2_O_2_). Drop test growth assays showed that besides glycerol and H_2_O_2_, VvXIP1 also transports boric acid, copper, arsenic and nickel. Furthermore, we found that *VvXIP1* transcripts were abundant in grapevine leaves from field grown plants and strongly repressed after the imposition of severe water-deficit conditions in potted vines. The observed downregulation of *VvXIP1* expression in cultured grape cells in response to ABA and salt, together with the increased sensitivity to osmotic stress displayed by the *aqy*-null yeast overexpressing *VvXIP1*, corroborates the role of VvXIP1 in osmotic regulation besides its involvement in H_2_O_2_ transport and metal homeostasis.

## Introduction

Plants display a much larger diversity of MIPs than other organisms, with 35 isoforms in Arabidopsis, 55 in poplar, 71 in cotton and at least 36 in maize [1]. Twenty-three different isoforms were found in the genome of the evolutionarily early land plant *Physcomitrella patens* [2]. The MIP superfamily in plants was initially divided into four subfamilies: the Plasma Membrane Intrinsic Proteins (PIPs), Tonoplast Intrinsic Proteins (TIPs), Nodulin-like Intrinsic Proteins (NIPs) and the Small Basic Intrinsic Proteins (SIPs). More recently, three new subfamilies were identified: the GlypF-like Intrinsic Proteins (GIPs) and the Hybrid Intrinsic Proteins (HIPs) in *P*. *patens*, and the Uncharacterized Intrinsic Proteins (XIPs) in a number of dicotyledonous plants, including tomato and grapevine [3,4], but not in Arabidopsis [5].

The most conserved feature of XIP protein sequences, which can be used as a signature of this subfamily, is the NPARC motif, with a cysteine residue located after the second NPA motif [3,5–8]. In addition, XIPs show considerable amino acid variation at both the first NPA motif and the ar/R filter. Based on the four amino acids defining the ar/R filters, XIPs from eudicots can be divided into four subclasses, two of which have an ar/R signature similar to that found in some plant NIPs, while the other two are even more hydrophobic [2].

In grapevine, up until now, 23 MIPs were identified with genome sequence analysis in a single cultivar [6,9]. Several studies have linked AQP activity to the vine water status, but *V*. *vinifera* cultivars have different tolerance and responses to water deficit stress, and these differences are particularly significant between isohydric and anisohydric grapevines. While Chardonnay (anisohydric) exhibited a significant increase in *VvPIP1;1* expression under water stress, Grenache (isohydric) did not show any alteration [7]. MIP expression also changes during maturation of grapes, and this phenomenon is correlated with the increase in hydraulic resistance observed in the post-veraison stages [9].

In grapevine, we found a putative *XIP* gene that was cloned and its subcellular localization was assessed using Agrobacterium-mediated transformation of *Nicotiana benthamiana* epidermal cells. Subsequent studies were performed to functionally characterize VvXIP1 in yeast cells by stopped-flow spectroscopy and drop test growth assays. Results confirmed that VvXIP1 transports glycerol, H_2_O_2_, copper, boric acid, arsenic, but water transport could not be detected. Real-time PCR studies were performed in samples from field-grown plants, potted vines and cultured cells to understand where *VvXIP1* is expressed and how it is regulated. A new role for XIP proteins in the transport of H_2_O_2_ and heavy metals and metalloids at the ER membranes and its involvement in water-stress response is discussed.

## Materials and Methods

### *In silico* studies

Phylogenetic analysis was performed *in silico* using amino acid sequences from *Vitis vinifera* (F6I152), *Gossypium hirsutum* (D9DBX6), *Populus trichocarpa* (EEE86940), *Prunus persica* (M5VMI6), *Nicotiana tabacum* (ADO66667), *Lotus japonicus* (CCI69207), *Ricinus communis* (B9T717), *Physcomitrella patens* (XP_001758094), *Aspergillus terreus* (Q0CWK8), *Fusarium oxysporum* (N4U8H1), *Penicillium marneffei* (B6QIR3), *Hypocrea virens* (G9N6L5), *Hypocrea jecorina* (G9N6L5) and *Selaginella moellendorffii* (XP_002971714) obtained from the National Center of Biotechnology (NCBI), Uniprot and PlantGDB using the BLAST tool. The alignment of sequences was performed with PRANKSTER and Genedoc [10]. The phylogenetic tree was created using these alignments with PROTDIST, NEIGHBOR and RETREE from the PHYLIP software package [11] and Mega 4 [12]. An alignment with VvXIP1 and several plant XIPs was performed and TOPCONS [13] was used to identify the transmembrane helix domains.

### Plant material

Grape berries, canes, flowers and leaves of cv. Vinhão were collected from a commercial vineyard near Guimarães, Portugal (41°25'16.6N 8°14'38.4W) with permission from the owner of the field. Potted cv. Aragonez plants were grown in a greenhouse and subjected to different watering regimes during 4 weeks: full irrigation (FI–control), with plant watering every two days; and non-irrigation (NI), without watering. Leaves were collected when NI plants’ water potential was -1.3 < Ψpd < -0.9 MPa [14].

Cell suspensions of *V*. *vinifera* L. (Cabernet Sauvignon Berry—CSB) were freshly established from somatic callus that was previously initiated from Cabernet Sauvignon berry pulp. They were maintained on modified Murashige and Skoog (MS) medium, supplemented with 2% (w/v) sucrose in 250 mL flasks on a rotary shaker at 100 rpm in the dark, at 23°C according to [15]. Cells were subcultured weekly by transferring 10 mL aliquots into 40 mL of fresh medium. To study the effect of different treatments on *VvXIP1* expression, 5 mL aliquots were incubated overnight with 100 mM NaCl, 150 μM abscisic acid (ABA), 150 μM salicylic acid (SA), 100 μM copper sulfate (CuSO_4_) and 100 μM boric acid (H_3_BO_3_) at 23°C. After each treatment, cells were immediately frozen in liquid nitrogen and stored at -80°C.

### Yeast strains and growth conditions

The *Saccharomyces cerevisiae* strains YSH1172 (*MATα leu2*::*hisG trp1*::*hisG his3*::*hisG ura3-52 aqy1*::*KanMX4 aqy2*::*HIS3;* [16]) and CEN.PK113-5D (*MATα MAL2-8c SUC2 ura3-52*) were used in this study. YSH1172 is a deletion mutant for aquaporins and was transformed with *pVV214-VvXIP1* and *pVV214-Empty Vector*. CEN.PK113-5D was used to study the subcellular localization of VvXIP1 in yeast after overexpressing *VvXIP1-GFP*. The constructs were prepared using Gateway (Invitrogen; primers are found in S1 Table) and introduced in the cells by the LiAc/SS-DNA/PEG method [17]. YSH1172 strain was grown in synthetic medium containing 7 g L^-1^ YNB, 1.3 g L^-1^ SC, 2% glucose, 30 mg L^-1^ leucine and 25 mg L^-1^ tryptophan. CEN.PK113-5D strain was grown in synthetic medium containing 7 g L^-1^ YNB, 1.3 g L^-1^ SC and 2% glucose.

### Metal and metalloid toxicity tests

To test metal sensitivity, YSH1172 *S*. *cerevisiae* strain overexpressing *VvXIP1* was cultivated in media supplemented with toxic levels of heavy metals and metalloids. Yeast cells were grown to a final OD_600 nm_ = 1, different serial dilutions (0.1, 0.01 and 0.001) were made and 10 μL from each suspension were spotted in solid YNB medium supplemented with: i) 40 mM boric acid (adjusted to pH 5.5 with Tris); ii) 5 mM CuSO_4_; iii) 0.45 mM arsenic acid; iv) 0.5 mM nickel chloride. Appropriate control plates inoculated with non-complemented mutants were also prepared.

### Growth experiments with H_2_O_2_, glycerol or sorbitol

To confirm the capacity of VvXIP1 to transport glycerol and H_2_O_2_ the *S*. *cerevisiae* strain YSH1172 overexpressing *VvXIP1* was grown to log phase, as described above. Different serial dilutions (0.1, 0.01 and 0.001) were spotted in solid YNB medium supplemented with: i) 0.35 and 0.7 mM H_2_O_2_ and ii) 1% and 2% of ethanol/glycerol. To study the role of VvXIP1 in osmotic stress response the yeast strain was cultivated on solid medium with 2.1 M sorbitol.

### Fluorescence assays to measure H_2_O_2_ uptake

The YSH1172 *aqy*-null *S*. *cerevisiae* strain transformed with *pVV214-VvXIP1* and *pVV214-empty vector* (control) constructs were used to study the transport of H_2_O_2_. Liquid cultures were grown overnight in the dark in presence of 1 μM 5-(and-6)-chloromethyl-2’,7’-dichlorodihydrofluorescein diacetate acetyl ester (CM-H_2_DCFDA, Molecular Probes), a fluorophore sensitive to reactive oxygen species (ROS) [18]. In its acetylated form, the dye can freely diffuse into yeast cells but, once inside, the fluorochrome is deacetylated and unable to cross the membrane making it susceptible to oxidation by ROS. After incubation, cells were washed three times with MOPS buffer (pH 7.0) and resuspended in this buffer to a final OD_600 nm_ = 1.4. After the addition of 50 μM H_2_O_2_, the fluorescence of 2 mL yeast suspension was followed over time at 20°C in a spectrofluorometer at an excitation/emission of 492/527 nm (Perkin Elmer Luminescence Spectrometer LS 50).

### Clark electrode assays

The YSH1172 *aqy*-null yeast strain was transformed with *pVV214-VvXIP1* and *pVV214-empty vector* (control) constructs and pre-cultured in YNB+SC solid medium. Liquid cultures were then grown overnight. Cells were then washed three times and resuspended in water to a final OD_600 nm_ = 1.0. H_2_O_2_ was then added to the cell suspension to a final concentration of 50 μM and the O_2_ formation was followed with a Clark electrode coupled to an YSI 5300 Biological Oxygen Monitor. The rate of O_2_ release by the yeast strain overexpressing *VvXIP1* was compared with control cells and used as a measure of H_2_O_2_ uptake. To evaluate the sensitivity of VvXIP1 to mercury, 1 mM HgCl_2_ was added to the reaction chamber before or after the addition of H_2_O_2_.

### RNA isolation from grapevine berries and leaves

RNA from grape berries and leaves was isolated with a QIAGEN RNeasy Plant Mini kit following an adaptation of the manufacturer’s instructions. The extraction buffer was changed to 2% cetyltrimethyl ammonium bromide (CTAB), 2% soluble polyvinylpyrrolidone (PVP) K-30, 300 mM Tris-HCl (pH 8.0), 25 mM EDTA, 2.0 M NaCl, and 2% (v/v) β-mercaptoethanol. After an in-column DNase treatment, the RNA integrity was checked in a 1% agarose gel, and the first-strand cDNA synthesis was performed with the LongRange 2Step RT-PCR (Qiagen), following manufacturer’s instructions.

### Subcellular co-localization studies

The *pH7RWG2-VvXIP1-RFP* construct was prepared following the Gateway technique (Invitrogen) with the primers shown in S1 Table. Recombination sequences were introduced by PCR in the *VvXIP1* cDNA without a stop codon and the fragment recombined into the entry vector pDONR221 with the BP clonase enzyme. *VvXIP1* cDNA was then recombined into the pH7RWG2 vector by the LR clonase enzyme. The constructs *pCambia2300-YFP-ZmPIP2;5* and *pCambia2300-YFP-ZmTIP2;1*, *pCambia2300-YFP-HDEL* and *pCambia2300-ST-YFP* were obtained as previously described [19]. Constructs were separately introduced in *Agrobacterium tumefaciens* (GV3101) and transient transformation of *N*. *benthamiana* leaf epidermal cells was performed according to a previous report [20]. Bacterial cells were cultivated overnight in liquid LB medium up to the exponential-stationary phase and then diluted to OD_600nm_ = 0.1 with infiltration buffer (50 mM MES pH 5.6, 2 mM Na_3_PO_4_, 0.5% glucose, and 100 μM acetosyringone). Diluted cells were cultivated again until the culture reached an OD_600nm_ = 0.2. Four-week-old *N*. *benthamiana* plants were infiltrated with the bacterial cultures and leaf discs were examined under the confocal microscope 2 days later in a Leica TCS SP5IIE scanning confocal microscope (Leica Microsystems). Data stacks were analysed and projected using ImageJ 1.42m software (http://rsb.info.nih.gov/ij/). The yellow fluorescence signal from YFP was represented in green in all the acquisitions. The yeast CEN.PK113-5D was used to study the subcellular localization of VvXIP1-GFP with the epifluorescence microscope.

### Real-time PCR studies

Quantitative Real-time PCRs were performed with a QuantiTect SYBR Green PCR Kit (Qiagen) and in a CFX96 Real-Time detection System (Bio-Rad), at an annealing temperature of 55°C. RNA and cDNA samples were obtained as described above. Experiments were carried out in biological triplicates using *VvGAPDH* as an internal control. To verify the absence of unspecific and primer-dimer amplification, melting curves were performed after each run. Data were analyzed using gene expression tool in the CFX Manager Software 2.0 (Bio-Rad). The primers used to study the expression of *VvXIP1* and *VvGAPDH* are described in S1 Table.

### Isolation of yeast subcellular membranes

YSH1172 *S*. *cerevisiae* cells transformed with *pVV214-VvXIP1* and *pVV214-empty vector* (control) were cultivated overnight in YNB medium with leucine and tryptophan. Cells were washed, resuspended in digestion buffer (1.35 M sorbitol, 10 mM citric acid, 30 mM Na_2_HPO_4_, 1 mM EGTA, pH 7.4) with 30 mM dithiothreitol (DTT), centrifuged and spheroplasts were obtained by digestion with zymolyase 20T (in digestion buffer). After complete digestion, monitored in a phase contrast microscope, spheroplasts were pelleted and homogenized in a potter homogenizer after resuspension in HEPES-lysis buffer [20 mM HEPES, 50 mM potassium acetate, 100 mM sorbitol, 2 mM EDTA, 1 mM phenylmethylsulphonyl fluoride (PMSF), 1 mM DTT, pH 6.8]. The homogenate was centrifuged at 1000 *g* for 10 min and the supernatant saved. The membranes were then pelleted at 100.000 *g* for 30 min, resuspended (100 mM mannitol, 10 mM Tris-HEPES, pH 7.5), centrifuged again at 100.000 *g* for 30 min, flash frozen, and stored at -80°C. These procedures were performed as described previously [8,21].

### Stopped flow spectroscopy to measure water and glycerol transport

Membrane permeability of the microsomal fraction from transformed yeasts was studied by the stopped flow technique (HI-TECH Scientific PQ/SF-53). Five runs were usually stored and analysed in each experimental condition, as described before [8,22]. To assess water transport, vesicles resuspended in 100 mM mannitol, 10 mM Tris-HEPES (pH 7.5) (0.1 ml, 0.4 mg protein mL^-1^) were mixed with a solution containing 340 mM mannitol and 10 mM Tris-HEPES (pH 7.5) at 23°C to produce an inwardly directed gradient of impermeant solute (osmotic gradient 240 mOsM). The kinetics of vesicle shrinkage was measured from the time course of 90° scattered light intensity at 400 nm until a stable light scatter signal was reached. Experiments were performed at 14–33°C to evaluate the activation energy of water transport (*Ea*).

To assess glycerol transport, vesicles resuspended in 100 mM mannitol, 10 mM Tris-HEPES (pH 7.5) (0.1 mL, 0.4 mg protein mL^-1^) were mixed with a solution containing 100 mM mannitol, 240 mM glycerol and 10 mM Tris-HEPES (pH 7.5) at 23°C to produce an inwardly directed gradient (osmotic gradient 240 mOsM). After a fast shrinkage due to water outflow, the kinetics of vesicle swelling due to glycerol influx (with consequent water influx) was measured from the time course of 90° scattered light intensity at 400 nm until a stable light scatter signal was reached. The water permeability coefficient (*P*_*f*_) and the glycerol permeability coefficient (*P*_*gly*_) were estimated by fitting the light scatter signal to a single exponential curve with the equation *P*_*f*_ = *k*(*V*_*0*_/*A*) [1/*V*_*w*_/(osm_out_)_∞_] [23], where *V*_*w*_ is the molar volume of water, *V*_*0*_/*A* is the initial volume to area ratio of the vesicle population and (osm_out_)_∞_ is the final medium osmolarity after the application of the osmotic gradient. The osmolarity of each solution was determined from freezing point depression by a semi-micro-osmometer (Knauer GmbH, Germany). The activation energies (*Ea*) were obtained from the slope of an Arrhenius plot (ln *P*_*f*_ or ln *P*_*gly*_ as a function of 1/T) multiplied by the gas constant R. Vesicle size (initial volume) was determined by quasi-elastic light scattering (QELS) by a particle sizer (BI-90 Brookhaven Instruments) as described in [23].

### Statistical analysis

The results obtained were statistically verified by analysis of variance tests (one-way and two-way ANOVA using Prism v. 6 (GraphPad Software, Inc.) Post-hoc multiple comparisons were performed using the HSD Tukey test.

## Results

### *VvXIP1* sequence analysis and subcellular localization

Phylogenetically, VvXIP1 is particularly close to GhXIP1 from cotton and to PpXIP1 from peach among XIP members from different plant and fungi species (S1 Fig). XIPs proteins share the highly conserved NPARC motif, despite slight variations in the first NPA, which is NPV in most of the aligned species. Using TOPCONS it was also possible to identify regions corresponding to the six transmembrane helixes (S2 Fig).

**Fig 1:**
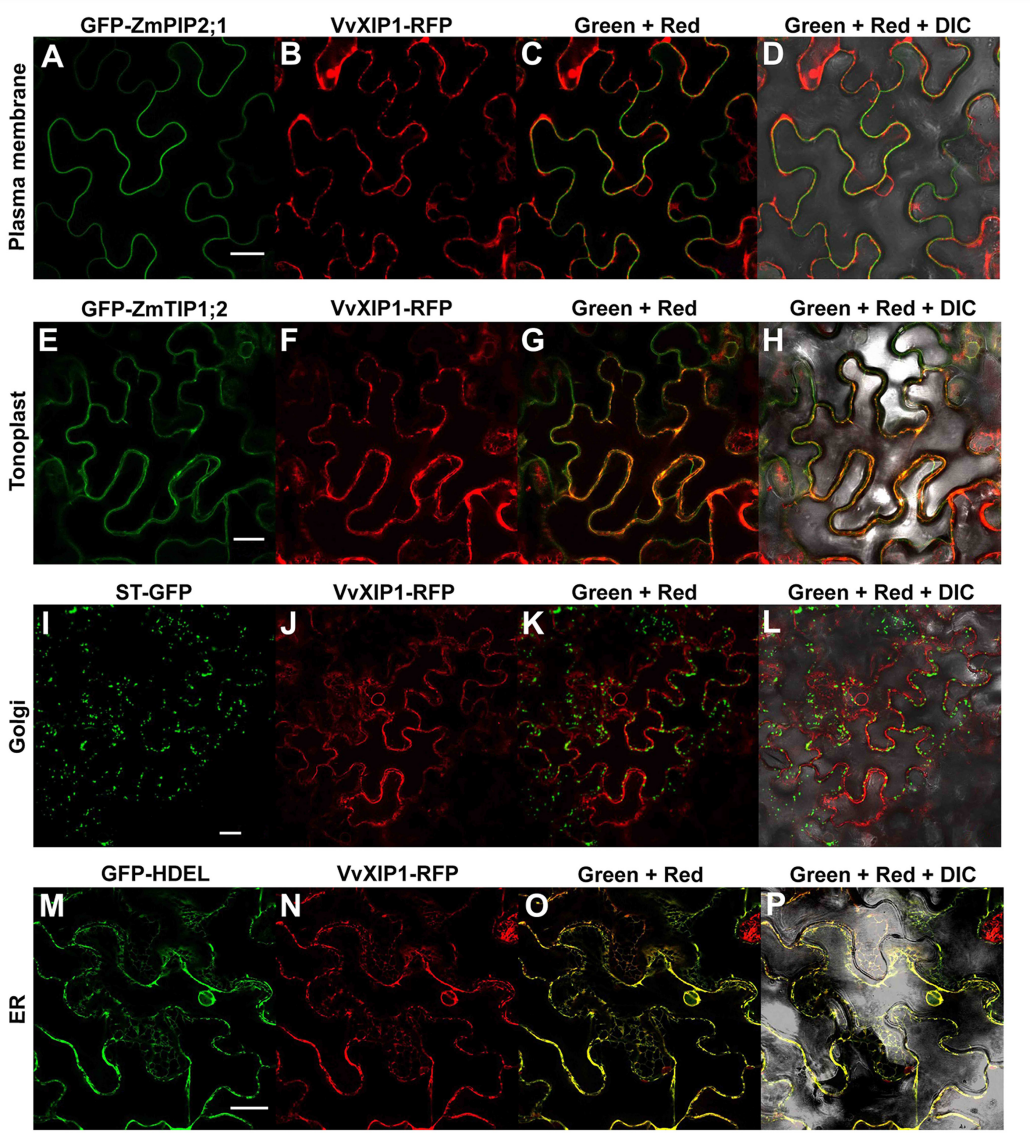
Subcellular localization studies of VvXIP1-RFP in tobacco epidermal cells (A-P). VvXIP1-RFP was co-transformed with YFP-ZmPIP2;5 (plasma membrane marker, A-D), YFP-TIP1;2 (tonoplast marker, E-H), ST-YFP (Golgi marker, I-L), and YFP-HDEL (ER marker, M-P).

To study the subcellular localization of VvXIP1, the corresponding XIP1-RFP fusion proteins were transiently expressed in *N*. *benthamiana* epidermal cells. Co-localization studies with the fusion proteins YFP-PIP2;5 (plasma membrane marker), YFP-HDEL (ER marker), ST-GFP (Golgi marker), YFP-ZmTIP2;1 (tonoplast marker) and VvXIP1-RFP revealed that VvXIP1 is abundant at the ER membrane but some labeling of the tonoplast was also observed (Fig 1). S3 Fig shows a more conspicuous labeling pattern of the ER in different epidermal cells of *N*. *benthamiana*. In yeasts overexpressing *VvXIP1-GFP*, the green fluorescence signal was abundant in the ER but other membranes were also labeled, as shown before when yeasts overexpress other intracellular proteins (S4 Fig, [24]).

### VvXIP1 transports glycerol but not water

**Fig 2:**
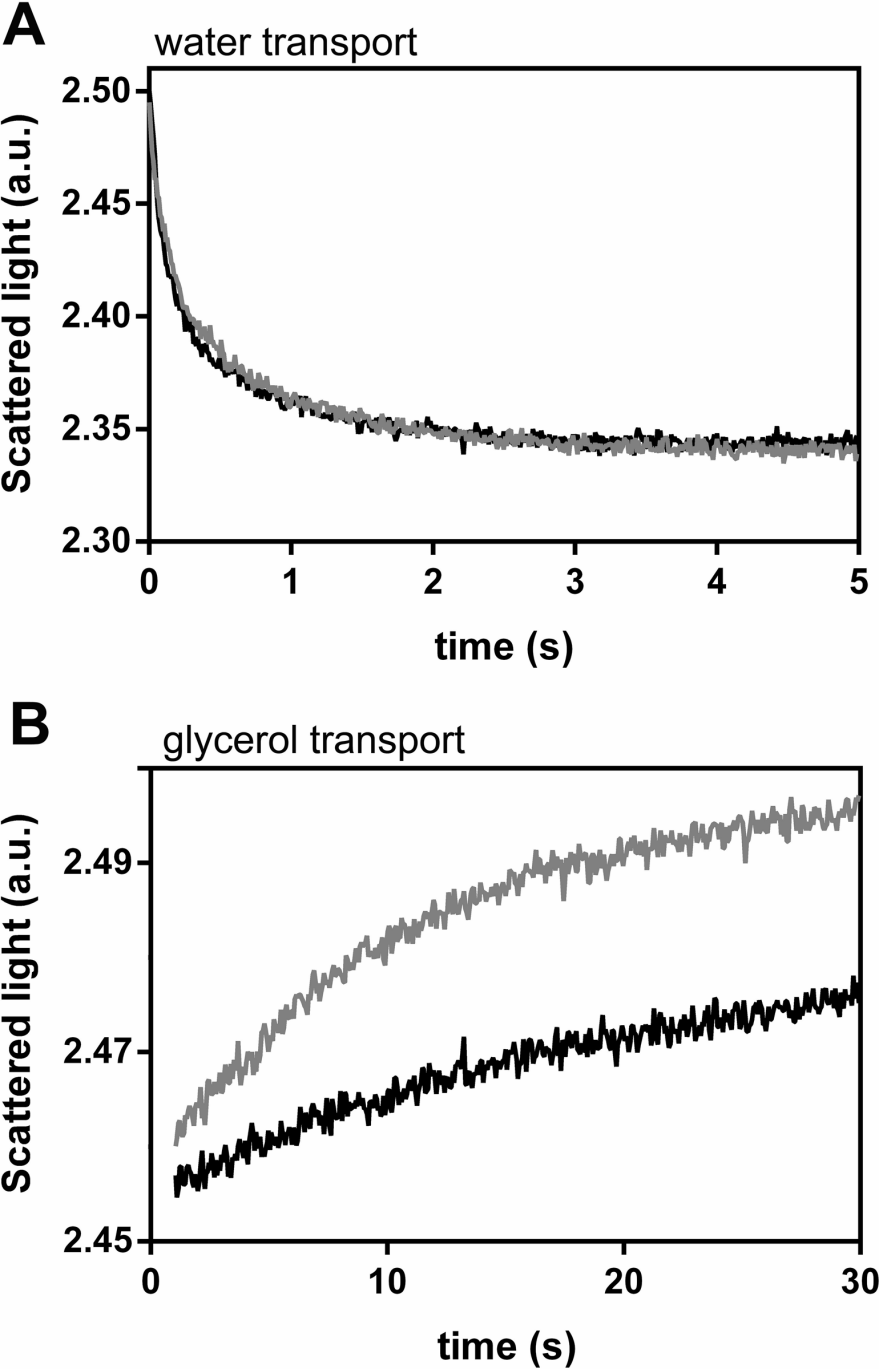
Study of VvXIP1 water and glycerol permeability in yeast. Normalized scattered light intensity obtained from stopped flow experiments performed at 28°C with membrane vesicles collected from yeast cells transformed with *pVV214-VvXIP1* (grey) or the empty vector (black), suddenly exposed to an osmotic gradient of 240 mOsM with a mannitol solution (A) and with a glycerol solution (B).

Membrane permeability by stopped flow light-scattering spectrophotometry was measured in crude membrane fractions isolated from the yeast strain YSH1172 (*aqy-*null) transformed with *pVV214-VvXIP1*. Vesicle size, measured by QELS, was homogeneous in all batches with a mean hydrodynamic diameter of 375 ± 62 nm (n = 8). No differences in the rates of water efflux were observed (Fig 2A) when control and *VvXIP1*-expressing vesicles were challenged with a hypertonic mannitol solution resulting in similar osmotic permeability coefficient (*P*_*f*_; Table 1). Likewise, the activation energies (*Ea*) determined in both vesicle batches show similar values (Table 1; S5 Fig).

**Table 1:** Permeability (*P*_*f*_ and P_*gly*_) and activation energy (*Ea*) for water transport in yeast membranes obtained by stopped flow (for details see Material and Methods).

		Permeability (cm s^-1^)	*Ea* (kcal mol^-1^)
**Water *P***_***f***_ **(x10**^**3**^**)**	Empty vector	4.31 ± 0.1	9.7 ± 0.95
	*VvXIP1*	3.70 ± 0.1	9.1 ± 0.68
**Glycerol P**_**gly**_ **(x10**^**5**^**)**	Empty vector	9.62 ± 1.0	26.0 ± 1.14
	*VvXIP1*	40.42 ± 2.0*	11.8 ± 0.76

Asterisks denote significant differences compared to the control

* = P ≤ 0.01.

When the yeast membrane vesicles were suddenly exposed to an osmotic gradient of 240 mOsM with a glycerol solution, results showed that VvXIP1 facilitated glycerol uptake that, in turn, promoted a higher rate of swelling in vesicles from VvXIP1-expressing yeast cells due to an increased osmotic water movement (Fig 2A). The corresponding permeability coefficients are shown in Table 1. The *Ea* value associated with glycerol transport in vesicles from VvXIP1-expressing yeast cells was much lower than in vesicles from the yeast transformed with the empty vector, indicating protein-mediated glycerol permeation (Table 1, S5 Fig).

Moreover, yeasts overexpressing *VvXIP1* grew better than control cells (transformed with the empty vector) in solid media supplemented with 1% ethanol and glycerol, confirming that glycerol is a substrate of VvXIP1 (S6 Fig). Altogether, stopped flow experiments with purified membrane vesicles and drop test assays with intact yeasts strongly suggested that VvXIP1 is unable to facilitate water diffusion but facilitates glycerol permeation efficiently.

### VvXIP1 transports H_2_O_2_

**Fig 3:**
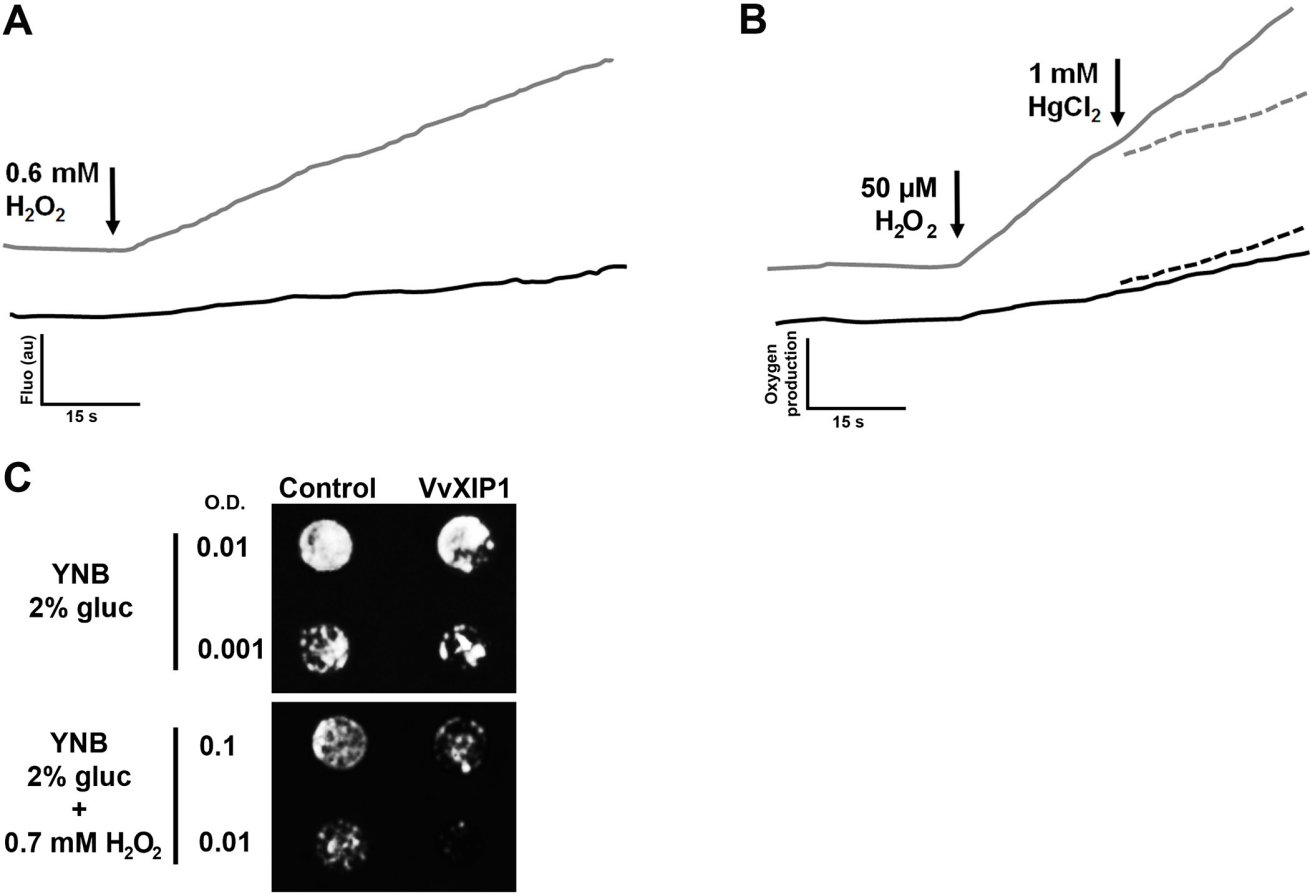
H_2_O_2_ transport by yeast cells expressing *VvXIP1*. Yeast cultures transformed with *pVV214-VvXIP1* (black line) and control cells (empty vector, grey line) were incubated overnight with the CM-H_2_DCFDA probe and fluorescence increase after the addition of 0.6 mM H_2_O_2_ was monitored in a spectrofluorimeter (A). O_2_ release (after the intracellular breakdown of H_2_O_2_) by cells transformed with *pVV214-VvXIP1* (black) and control yeast (grey) monitored with a Clark electrode in response to 50 μM H_2_O_2_ (solid lines) and after mercury inhibition (dashed lines) (B). Plate growth assays in YNB media supplemented with 0.6 mM H_2_O_2_ (C).

H_2_O_2_ transport was tested through different approaches in the yeast strain *aqy*-null overexpressing *VvXIP1*. When the probe CM-H_2_DCFDA was used to measure H_2_O_2_ uptake by spectrofluorometry, yeast cells expressing *VvXIP1* showed a much higher rate of fluorescence increase after H_2_O_2_ addition than control cells transformed with the empty vector (Fig 3A). In agreement, after addition of 50 μM H_2_O_2_ *VvXIP1*-transformed yeast showed a 3.5-fold higher rate of O_2_ release (produced after intracellular breakdown of H_2_O_2_) than the control, as measured with a Clark electrode (Fig 3B), but this increment was prevented by the addition of 1 mM HgCl_2_ (Fig 3B, dashed lines), suggesting that mercury completely inhibited H_2_O_2_ transport.

Accordingly, the growth of the transformed yeast was impaired when YNB media was supplemented with 0.7 mM H_2_O_2_ (Fig 3C). Altogether, the results in intact yeast cells with the H_2_O_2_-sensitive probe CM-H_2_DCFDA and with the oxygen electrode, together with the observed increased sensitivity of the transformed yeast to H_2_O_2_ toxicity strongly suggested that VvXIP1 facilitates the permeation of H_2_O_2_.

### Toxicity tests support that VvXIP1 transports heavy metals and metalloids

**Fig 4:**
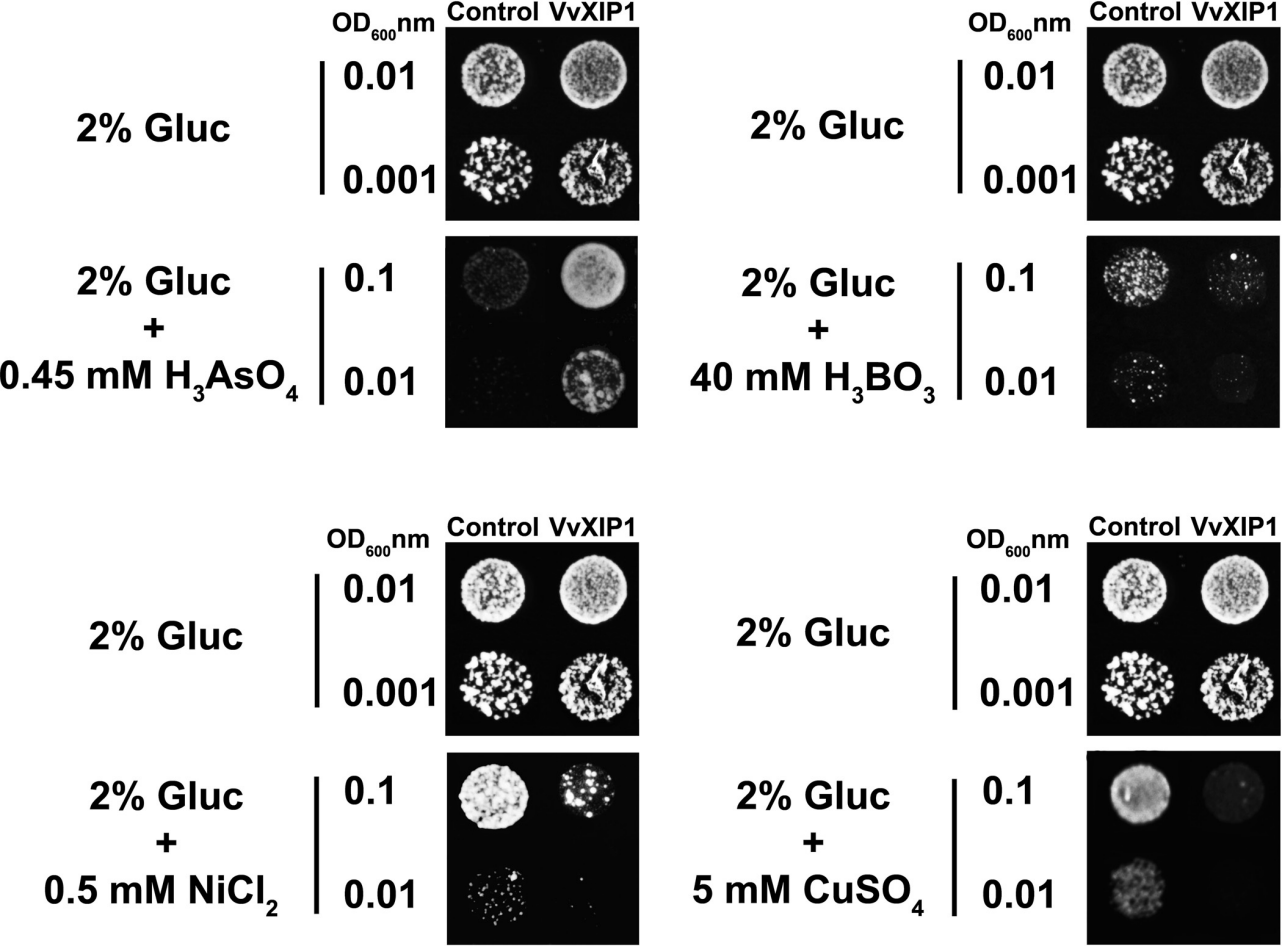
The effect of metals and metalloids in the growth of *S*. *cerevisiae* overexpressing *VvXIP1*. Yeast transformed with *pVV214-VvXIP1* and control cells (empty vector) were diluted (OD_600_ = 0.1, 0.01 and 0.001) and spotted on medium containing 0.45 mM H_3_AsO_4_, 40 mM H_3_BO_3_, 0.5 mM NiCl_2_ and 5 mM CuSO_4_ and the growth was recorded following 3–5 days at 30°C.

To evaluate the capacity of VvXIP1 to facilitate the transport of boron, arsenic, nickel and copper, the *aqy*-null mutant YSH1172 overexpressing *VvXIP1* was cultivated in solid media supplemented with toxic concentrations of these heavy metals and metalloids. Control growth experiments were performed with yeasts transformed with the empty vector. Results showed that the overexpression of *VvXIP1* significantly impaired the growth of the yeast cells in the presence of 40 mM boron, 0.5 mM nickel and 5 mM copper (Fig 4). Contrarily, the growth of the transformed yeast was improved in the presence of 0.45 mM arsenic, in agreement with previous results [25]. As a whole, these results are consistent with the capacity of VvXIP1 to facilitate the transport of heavy metals and metalloids.

### *VvXIP1* transcription is abundant in leaves and is repressed by drought stress

**Fig 5:**
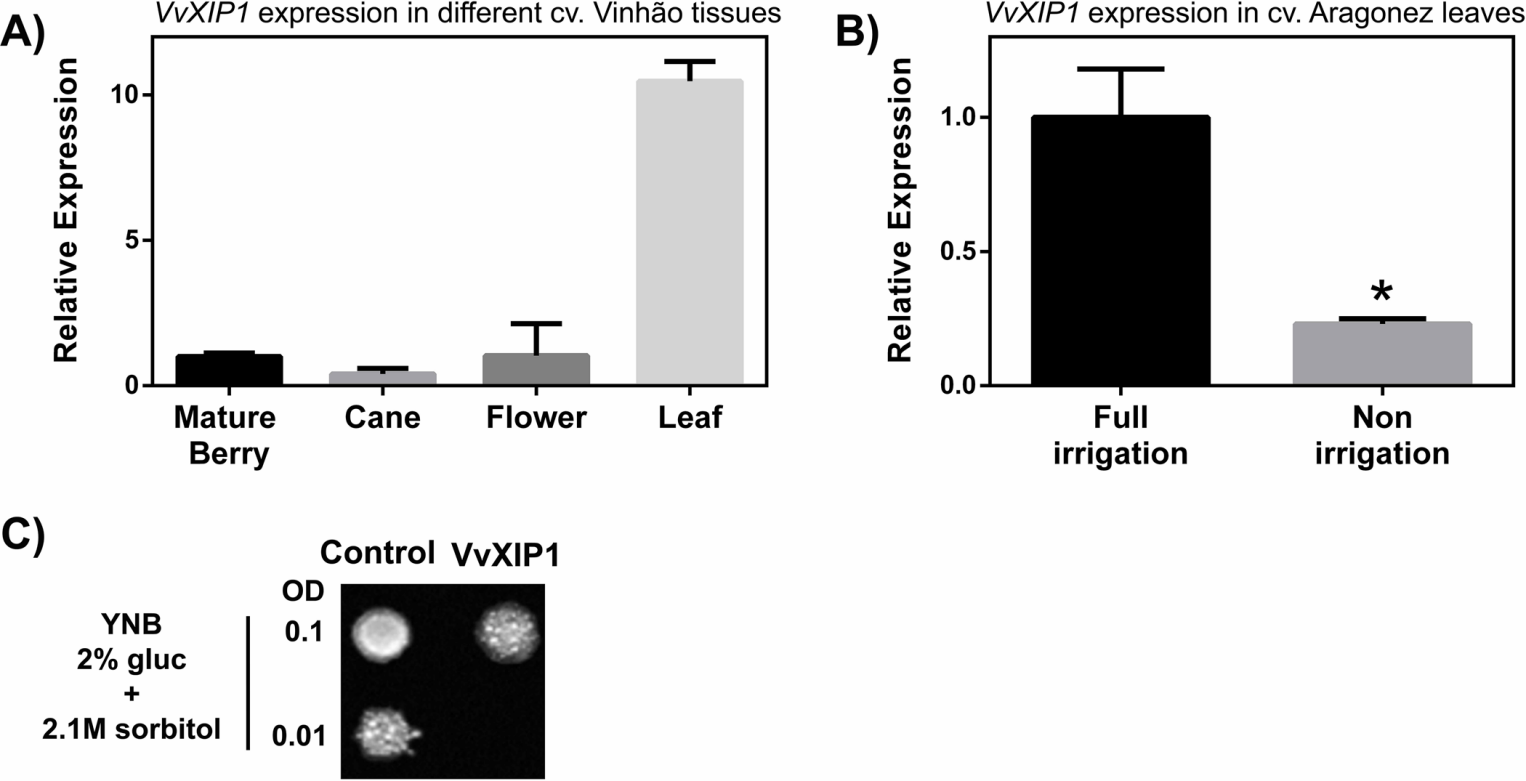
Study of *VvXIP1* expression in berries, canes, flowers and leaves from grapevine cv. Vinhão (A) grown under field conditions. *VvXIP1* steady-state transcript levels in leaves from potted grapevines (cv. Aragonez) under drought stress (B) and sorbitol induced osmotic stress sensitivity of yeast cells expressing *VvXIP1* (C).

Total RNA was isolated from leaves, mature berries, canes and flowers of field grown grapevines (cv. Vinhão), and qRT-PCR showed that *VvXIP1* transcripts were detected in all tested tissues but its steady-state expression was much higher in the leaves (Fig 5). Recently, when we studied the metabolic adjustments of grapevine berries and leaves in response to drought, we developed a standardized protocol to induce severe water stress in potted cv. Aragonez plants [14]. This approach allowed us to observe that the leaf expression of *VvXIP1* sharply decreases from control (full irrigated) to water-stressed plants (non-irrigated, Fig 5B). Furthermore, yeast cells overexpressing *VvXIP1* displayed an increased osmotic sensitivity in a hyperosmotic medium (+ 2.1 M sorbitol; Fig 5C), suggesting a role for VvXIP1 in the adjustment of grapevine osmotic stress response, as observed before for other aquaporins [16,22], in spite of VvXIP1 being unable to transport water.

### ABA, salt and copper downregulate *VvXIP1* transcription in CSB cells

**Fig 6:**
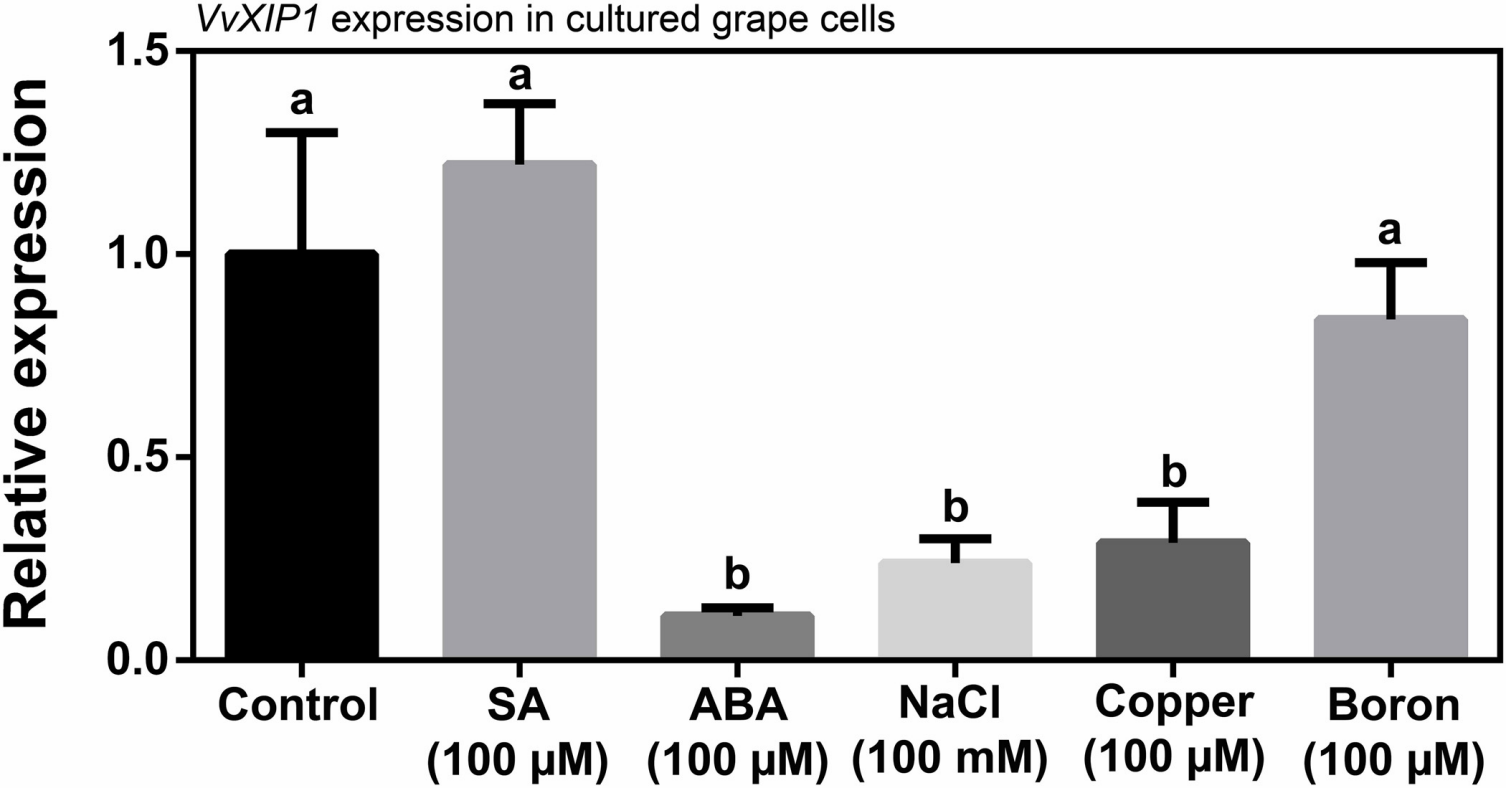
*VvXIP1* expression in grape cultured cells in response to hormones, salt, copper and boron. CSB cells were incubated overnight with SA (150 μM), ABA (150 μM), NaCl (100 mM), copper (100 μM) and boron (100 μM), and the expression of *VvXIP1* was studied by qRT-PCR.

Cultured grape cells (CSB) were used to study *VvXIP1* expression in response to an overnight incubation with 150 μM ABA, 150 μM SA, 100 mM NaCl, 100 μM CuSO_4_ and 100 μM boric acid (Fig 6). *VvXIP1* transcription was downregulated by ABA (but not by SA) and NaCl, which is in agreement with the role of *VvXIP1* observed above in the adjustment of osmotic stress response. An overnight incubation with 100 μM CuSO_4_ also strongly repressed *VvXIP1* transcription in grape cultured cells, which goes in agreement with results from field-grown grapevines when the exogenous application of the Bordeaux mixture also repressed *VvXIP1* expression in leaves (S7 Fig).

## Discussion

### VvXIP1 is a grapevine solute channel abundant at the ER membrane

So far the Uncharacterized Intrinsic Proteins (XIPs) were studied in few plant species and fungi, thus the knowledge about their true physiological roles and localization at the cellular/tissue levels is still somewhat scarce. After their discovery at the plasma membrane of erythrocytes as water channels, aquaporins were also found widespread in intracellular membranes [26]. This is the well-studied case of TIPs, which are very abundant at the tonoplast and have key roles in water transport into and out of the vacuole [9]. Also, the grapevine small basic intrinsic protein VvSIP1 was shown to have intracellular water channel activity at the endoplasmic reticulum, following co-localization studies and its functional characterization after its purification to homogeneity and incorporation in phosphatidylethanolamine liposomes [8]. Up until now, only the subcellular localization of two XIP aquaporins, NtXIP1;1 (*Nicotiana tabacum*) and HbXIP2;1 (*Hevea brasiliensis*), has been performed, and both were ascribed to the plasma membrane [2,27]. In the present work, co-localization experiments in *N*. *bethamiana* leaves transiently expressing fluorescent-tagged proteins revealed that VvXIP1 may play an important role at the ER given its rather unusual substrate specificity.

### VvXIP1 transports glycerol, hydrogen peroxide but not water

The capacity of plant aquaporins to transport glycerol, boric acid, and H_2_O_2_, among other non-ionic small neutral solutes, like urea, is not new [28]. In mammals’ water-specific aquaporins, such as the prototypical AQP1, stringently exclude the passage of solutes, but point mutations in the ar/R filter allow passage of urea, glycerol, ammonia, and even protons, strongly suggesting that this constriction is a major checkpoint for solute permeability [29].

Here we found that VvXIP1 transports H_2_O_2_ in a mercury-sensitive manner (Fig 3). The binding mercury to SH-groups of cysteine residues, and most particularly a quite conserved cysteine at the vicinity of the first NPA repeat causes a steric inhibition in the protein reducing its transport activity [30]. H_2_O_2_ is an important molecule in plants with a dual function as it is both an oxidative stress inducer and a signaling molecule [31,32,33]. Future studies should address the challenging hypothesis that VvXIP1 plays a role in the efflux of H_2_O_2_ at the ER membrane that is produced in high amounts during disulfide bond formation resulting from protein folding mechanisms. Indeed, protein folding and generation of ROS as a byproduct of protein oxidation in the ER are closely linked events [34].

Intriguingly, although H_2_O and H_2_O_2_ are structurally and electrostatically similar, VvXIP1 was able to distinguish both substrates. Stopped flow scattered-light spectrophotometry in membrane vesicles from yeast overexpressing *VvXIP1* showed that their permeability to water was similar to control vesicles. Also, no changes were observed in *E*a of water transport across the membrane of the vesicles, supporting that VvXIP1 is unable to mediate water transport (Fig 2). This goes in agreement with previous observation that *Solaneaceae* XIPs [2] and also the HbXIP2;1 [27] do not show permeability for water, but transport many uncharged substrates, including glycerol, urea and boric acid. In contrast, PtXIP2;1 and PtXIP3;3 from *Populus trichocarpa* are water channels, although their water permeability is low when compared to members of the PIP subfamily [35]. Nevertheless, the possibility that VvXIP1 needs to interact with other aquaporins to function as a water channel, as previously showed in maize, cannot be excluded [4,19].

It has been shown that glycerol is a common substrate for non-water conducting MIPs, particularly from the NIP and XIP subfamilies [2]. But the physiological significance of glycerol transport through VvXIP1 deserves detailed investigation in future studies. Given the observed abundance of VvXIP1 at the ER it is tempting to suggest that it could play a role in glycerophospholipid biosynthesis or in the quality control pathway of misfolded and unassembled proteins, which can be assisted by glycerol as a chemical chaperone [36].

### VvXIP1 is involved in the transport of metalloids (boron and arsenic) and heavy metals (copper and nickel)

Specific MIPs within all domains of life mediate the transport of metalloids, including silicon, boron, arsenic and antimony, which are a group of biologically important elements ranging from the essential to the highly toxic [25]. Boron in plants participates in the organization of cell wall pectic polysaccharides, thus is involved in cell wall expansion [37,38]. The observed capacity of VvXIP1 to transport boron could mediate grapevine response to boron excess through its compartmentalization at the ER. At the physiological pH the most predominant form of boron is the undissociated boric acid (uncharged), which is known as the form that diffuses through aquaporins [39]. Besides boron, VvXIP1 was also able to mediate the transport of arsenic. Interestingly, the transformation of *S*. *cerevisiae* cells with *VvXIP1* improved its growth in media supplemented with 0.4 mM arsenic, suggesting that this aquaporin may be involved in detoxification mechanisms of this metalloid [25].

The first characterization of an aquaporin as a cation channel was not consensual [40], but the striking capacity of some aquaporins to mediate the transport of charged molecules is still being reported. This is the case of three tonoplast aquaporins (TIP) from *Triticum aestivum* that restored the ability of the yeast mutant Δ*mep1-3* deficient in ammonium transport to grow when 2 mM NH_4_^+^ was supplied as the sole N source [41]. In mammals, AQP1 was found to be a nonselective cation channel permeant to K^+^, Cs^+^, and Na^+^ and to a lesser degree to tetraethylammonium [40]. In the present study, results indicate that VvXIP1 facilitates the transport of the positively charged heavy metals nickel and copper. Future studies with yeast strains defective in copper transport [42–45] will allow to further elucidate the role of VvXIP1 in copper transport.

One important question remains regarding the path through which charged solutes like copper cross the aquaporin. It was suggested that AQP1 is permeated by water across the individual pores of each subunit of the tetramer, and the putative central pore lined by the four subunits facilitates the diffusion of charged solutes in response to cyclic nucleotide binding. In this regard, similarities between the carboxy tail domains of cyclic nucleotide gated (CNG) channels and AQP1 were found [46]. The observed capacity of VvXIP1 to transport charged solutes like heavy metals opens a new avenue of research on the role of aquaporins in response to heavy metal toxicity, and on its regulation at both transcriptional and protein activity levels.

### *VvXIP1* is downregulated by water-deficit stress

The observed high steady-state expression of *VvXIP1* in leaves from cv. Vinhão plants grown in the field suggested that this aquaporin could play an important role in this organ. Recently, it was showed that leaves and berries from water-stressed plants rearranged their metabolism to accumulate sugar-alcohols to cope with low water availability [14]. Here, using a similar approach, we found that *VvXIP1* is strongly downregulated in response to drought (Fig 6). In agreement, in CSB cultured cells, *VvXIP1* transcript levels were reduced by ABA and salt. The role of ABA on plant response to abiotic stresses, including water deficit and salt stress is well-known [9]. The treatment of maize roots with ABA results over 1–2 h in a transient increase in hydraulic conductivity of the whole organ and of cortical cells and also rapidly enhances the expression of some PIP isoforms [47]. In the present study we also showed that VvXIP1 greatly increased yeast cell sensitivity to water-deficit stress imposed by sorbitol, clearly confirming that VvXIP1 has an important role in osmotic regulation. Notably, under drought stress, osmolytes like polyols, glucose and fructose were found more abundant in leaves [14], which coincided with the observed downregulation of *VvXIP1*, supporting that it may be regulated by changes of the osmotic potential. Considering that this protein was not found to transport water, this effect could be due to its transporting capacity of osmotically active solutes, thus contributing to cell homeostasis.

## Supporting Information

S1 Fig Phylogenetic tree comparing XIP proteins from plants and fungi. Amino acid sequences from *Vitis vinifera* (Vv), *Populus trichocarpa* (Pt), *Prunus persica* (Pp), *Gossypium hirsutum* (Gh), *Ipomoea nil* (In), *Nicotiana tabacum* (Nt), *Lotus japonicus* (Lj), *Ricinus communis* (Rc), *Physcomitrella patens* (Ppat), *Aspergillus terreus* (Ate), *Fusarium oxysporum* (Fo), *Penicillium marneffei* (Pm), *Hypocrea virens* (Hv), *Hypocrea jecorina* (Hj) and *Selaginella moellendorffii* (Sm).
(DOCX)

S2 Fig Alignment of eight plant XIPs (VvXIP1, PtXIP1, PpXIP, GhXIP1;1, InXIP1;1 NtXIP1;1, LjXIP1 and RcXIP) showing six transmembrane helix domains (black lines) and the conserved ‘NPV’ and ‘NPARC’ motifs (grey lines). (DOCX)

S3 Fig Co-localization studies of VvXIP1-RFP with YFP-HDEL showing a strong overlapping of the fluorescence signals, indicating that this aquaporin is localized at the ER membrane. (DOCX)

S4 Fig Subcellular localization of VvXIP1-GFP in yeast cells. The yeast strain CEN.PK113-5D was transformed with the *VvXIP1-GFP* plasmid and observed under the epifluorescence microscope.
(DOCX)

S5 Fig Stopped flow experiment to evaluate the activation energy (*E*_a_) for water and glycerol transport in yeast vesicles. Normalized scattered light intensity was obtained from stopped-flow experiments performed according to a temperature gradient. Membrane vesicles purified from yeast cells transformed with *pVV214-VvXIP1* (grey) or the empty vector (black) were suddenly exposed to an osmotic gradient of 240 mOsM. The gradient was built with mannitol (A) to evaluate water transport and with glycerol (B) to evaluate glycerol transport.
(DOCX)

S6 Fig Yeast growth in glycerol media. Cultures of YSH1172 aqy-null yeast cells transformed with empty vector and *pVV214-VvXIP1* were spotted at OD_600_ nm of 0.1 0.01 on medium containing the indicated concentration of glycerol/ethanol and growth was recorded after 3 days at 30°C.
(DOCX)

S7 Fig Study of *VvXIP1* expression in leaves from grapevine cv. Vinhão (A) grown under field conditions and treated with copper in the form of Bordeaux misture.
(DOCX)

S1 Table Primer sequences used in this study. (DOCX)
