# Supplementary material for: The Grapevine Uncharacterized Intrinsic Protein 1 (VvXIP1) Is Regulated by Drought Stress and Transports Glycerol, Hydrogen Peroxide, Heavy Metals but Not Water
Source: PLoS One. 2016 Aug 9;11(8):e0160976. doi: 10.1371/journal.pone.0160976 (PMC4978503; doi:10.1371/journal.pone.0160976)
Supplement: S1 Fig — Amino acid sequences from Vitis vinifera (Vv), Populus trichocarpa (Pt), Prunus persica (Pp), Gossypium hirsutum (Gh), Ipomoea nil (In), Nicotiana tabacum (Nt), Lotus japonicus (Lj), Ricinus communis (Rc), Physcomitrella patens (Ppat), Aspergillus terreus (Ate), Fusarium oxysporum (Fo), Penicillium marneffei (Pm), Hypocrea virens (Hv), Hypocrea jecorina (Hj) and Selaginella moellendorffii (Sm). (DOCX) [file pone.0160976.s001.docx]

**S1 Figure.** Phylogenetic tree comparing XIP proteins from plants and fungi. Amino acid sequences from *Vitis vinifera* (Vv), *Populus trichocarpa* (Pt), *Prunus persica* (Pp), *Gossypium hirsutum* (Gh), *Ipomoea nil* (In), *Nicotiana tabacum* (Nt), *Lotus japonicus* (Lj), *Ricinus communis* (Rc), *Physcomitrella patens* (Ppat), *Aspergillus terreus* (Ate), *Fusarium oxysporum* (Fo), *Penicillium marneffei* (Pm), *Hypocrea virens* (Hv), *Hypocrea jecorina* (Hj) and *Selaginella moellendorffii* (Sm).
