# Supplementary material for: The Grapevine Uncharacterized Intrinsic Protein 1 (VvXIP1) Is Regulated by Drought Stress and Transports Glycerol, Hydrogen Peroxide, Heavy Metals but Not Water
Source: PLoS One. 2016 Aug 9;11(8):e0160976. doi: 10.1371/journal.pone.0160976 (PMC4978503; doi:10.1371/journal.pone.0160976)
Supplement: S2 Fig — (DOCX) [file pone.0160976.s002.docx]

**S2 Figure.** Alignment of eight plant XIPs (VvXIP1, PtXIP1, PpXIP, GhXIP1;1, InXIP1;1 NtXIP1;1, LjXIP1 and RcXIP) showing six transmembrane helix domains (black lines) and the conserved ‘NPV’ and ‘NPARC’ motifs (grey lines).

**
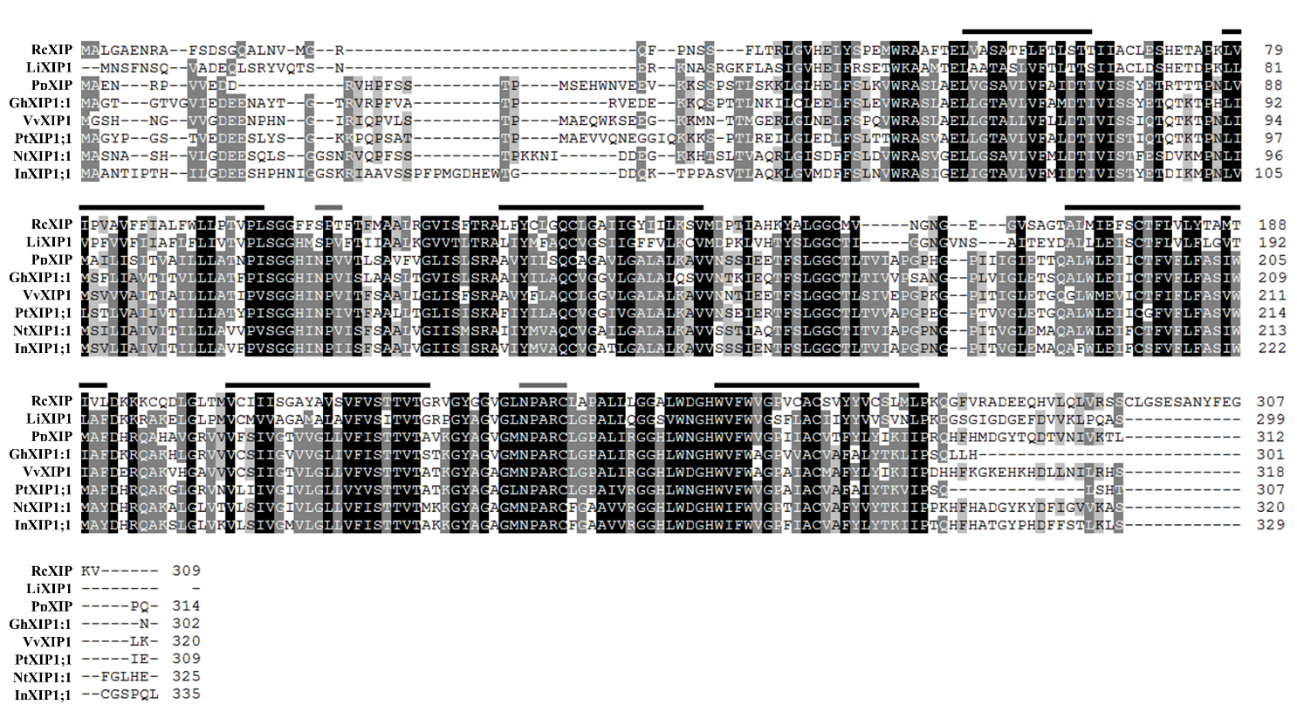
**
