# Supplementary material for: The Grapevine Uncharacterized Intrinsic Protein 1 (VvXIP1) Is Regulated by Drought Stress and Transports Glycerol, Hydrogen Peroxide, Heavy Metals but Not Water
Source: PLoS One. 2016 Aug 9;11(8):e0160976. doi: 10.1371/journal.pone.0160976 (PMC4978503; doi:10.1371/journal.pone.0160976)

**S3 Figure.** Co-localization studies of VvXIP1-RFP with YFP-HDEL showing a strong overlapping of the fluorescence signals, indicating that this aquaporin is localized at the ER membrane.


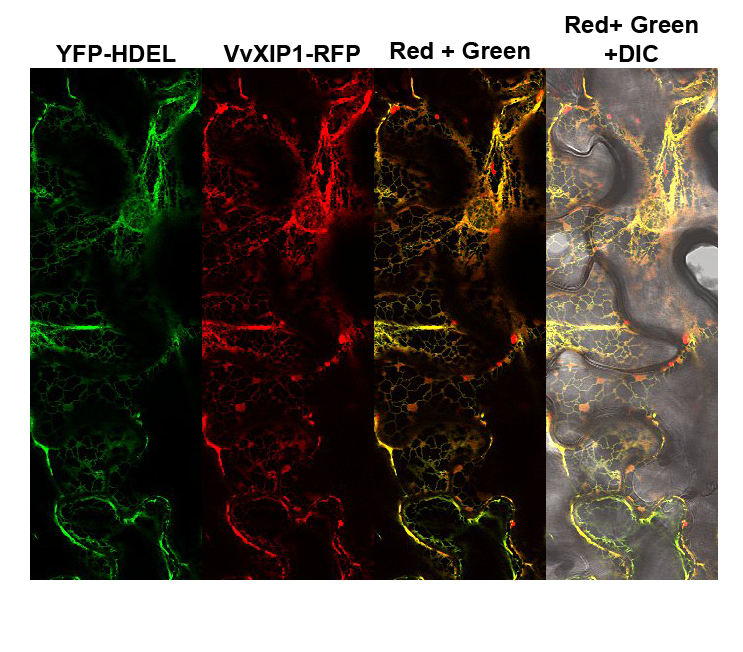

Supplement: S3 Fig — (DOCX) [file pone.0160976.s003.docx]
