# Supplementary material for: The Grapevine Uncharacterized Intrinsic Protein 1 (VvXIP1) Is Regulated by Drought Stress and Transports Glycerol, Hydrogen Peroxide, Heavy Metals but Not Water
Source: PLoS One. 2016 Aug 9;11(8):e0160976. doi: 10.1371/journal.pone.0160976 (PMC4978503; doi:10.1371/journal.pone.0160976)
Supplement: S4 Fig — The yeast strain CEN.PK113-5D was transformed with the VvXIP1-GFP plasmid and observed under the epifluorescence microscope. (DOCX) [file pone.0160976.s004.docx]

**S4 Figure.** Subcellular localization of VvXIP1-GFP in yeast cells. The yeast strain CEN.PK113-5D was transformed with the *VvXIP1-GFP* plasmid and observed under the epifluorescence microscope.

**
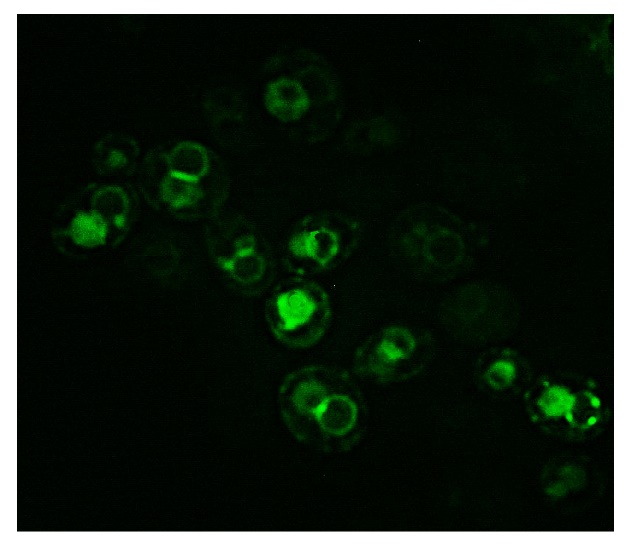
**
