# Supplementary material for: The Grapevine Uncharacterized Intrinsic Protein 1 (VvXIP1) Is Regulated by Drought Stress and Transports Glycerol, Hydrogen Peroxide, Heavy Metals but Not Water
Source: PLoS One. 2016 Aug 9;11(8):e0160976. doi: 10.1371/journal.pone.0160976 (PMC4978503; doi:10.1371/journal.pone.0160976)
Supplement: S5 Fig — Normalized scattered light intensity was obtained from stopped-flow experiments performed according to a temperature gradient. Membrane vesicles purified from yeast cells transformed with pVV214-VvXIP1 (grey) or the empty vector (black) were suddenly exposed to an osmotic gradient of 240 mOsM. The gradient was built with mannitol (A) to evaluate water transport and with glycerol (B) to evaluate glycerol transport. (DOCX) [file pone.0160976.s005.docx]

**S5 Figure.** Stopped flow experiment to evaluate the activation energy (*E*a) for water and glycerol transport in yeast vesicles. Normalized scattered light intensity was obtained from stopped-flow experiments performed according to a temperature gradient. Membrane vesicles purified from yeast cells transformed with *pVV214-VvXIP1* (grey) or the empty vector (black) were suddenly exposed to an osmotic gradient of 240 mOsM. The gradient was built with mannitol (A) to evaluate water transport and with glycerol (B) to evaluate glycerol transport.

**
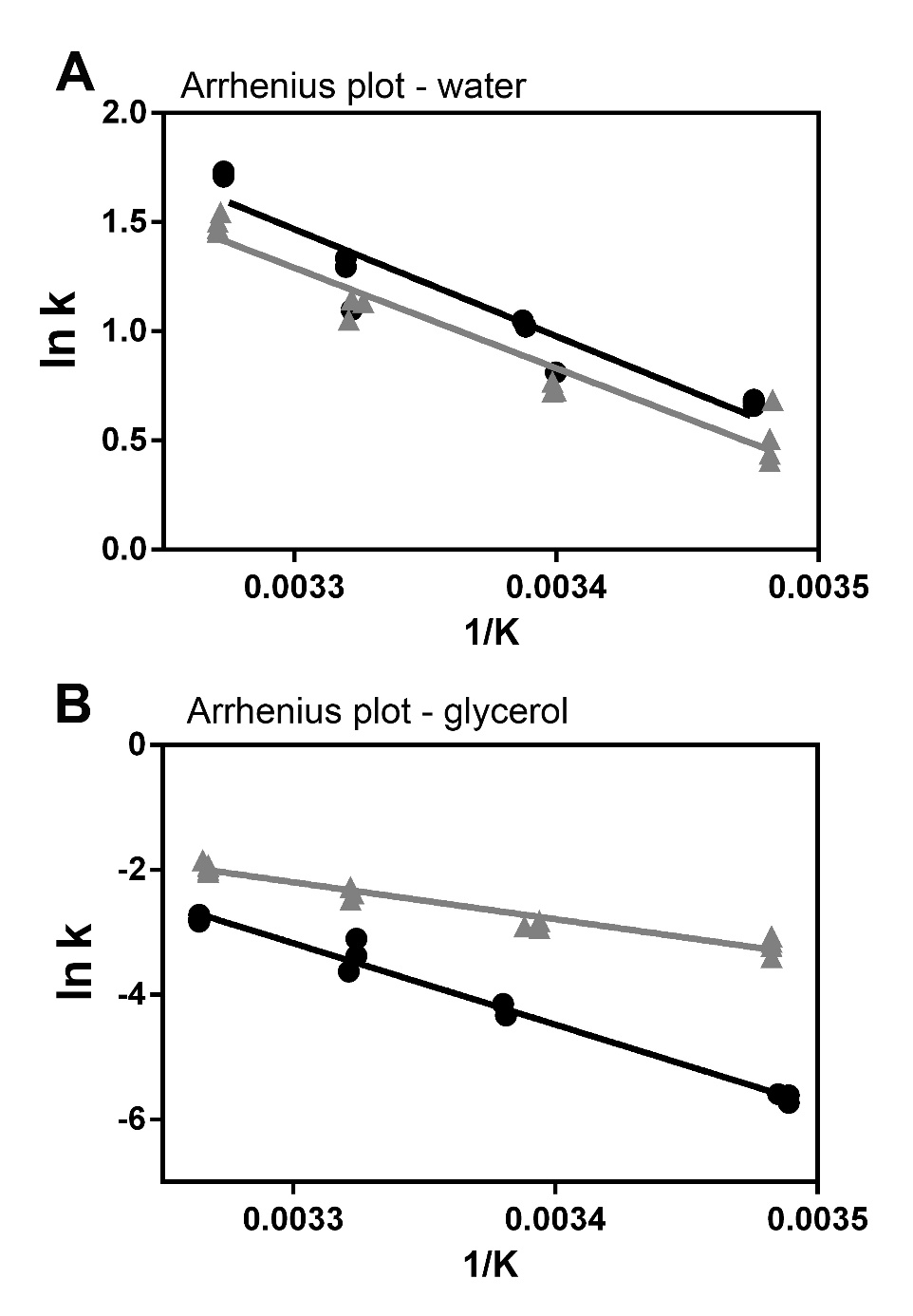
**
