# Supplementary material for: The Grapevine Uncharacterized Intrinsic Protein 1 (VvXIP1) Is Regulated by Drought Stress and Transports Glycerol, Hydrogen Peroxide, Heavy Metals but Not Water
Source: PLoS One. 2016 Aug 9;11(8):e0160976. doi: 10.1371/journal.pone.0160976 (PMC4978503; doi:10.1371/journal.pone.0160976)
Supplement: S6 Fig — Cultures of YSH1172 aqy-null yeast cells transformed with empty vector and pVV214-VvXIP1 were spotted at OD600 nm of 0.1 0.01 on medium containing the indicated concentration of glycerol/ethanol and growth was recorded after 3 days at 30°C. (DOCX) [file pone.0160976.s006.docx]

**S6 Figure.** Yeast growth in glycerol media. Cultures of YSH1172 aqy-null yeast cells transformed with empty vector and *pVV214-VvXIP1* were spotted at OD_600_ nm of 0.1 0.01 on medium containing the indicated concentration of glycerol/ethanol and growth was recorded after 3 days at 30 ºC.

**
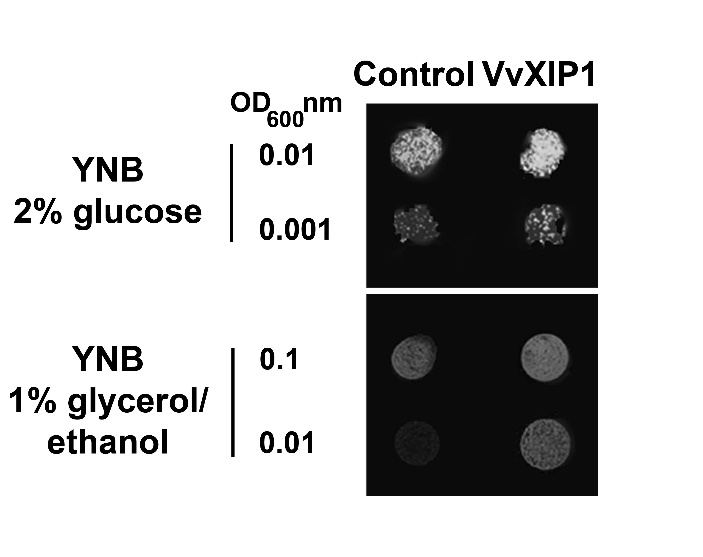
**
