# Supplementary material for: The Grapevine Uncharacterized Intrinsic Protein 1 (VvXIP1) Is Regulated by Drought Stress and Transports Glycerol, Hydrogen Peroxide, Heavy Metals but Not Water
Source: PLoS One. 2016 Aug 9;11(8):e0160976. doi: 10.1371/journal.pone.0160976 (PMC4978503; doi:10.1371/journal.pone.0160976)

**S7 Figure.** Study of *VvXIP1* expression in leaves from grapevine cv. Vinhão (A) grown under field conditions and treated with copper in the form of Bordeaux mixture.


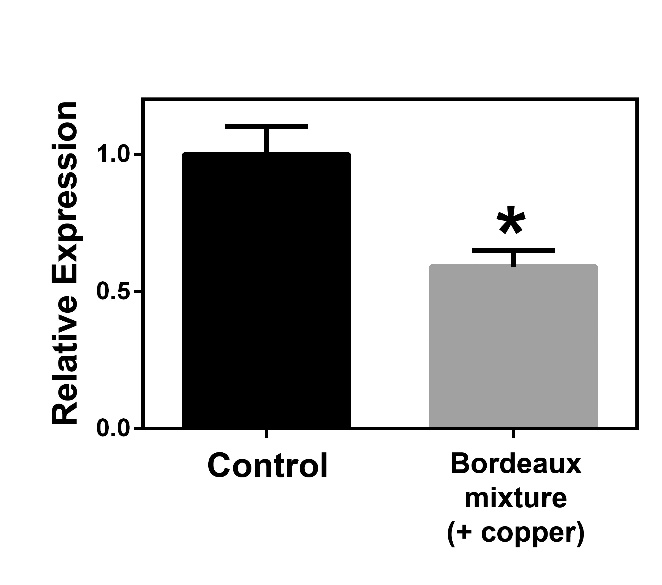

Supplement: S7 Fig — Vinhão (A) grown under field conditions and treated with copper in the form of Bordeaux misture. (DOCX) [file pone.0160976.s007.docx]
