# Supplementary material for: The Grapevine Uncharacterized Intrinsic Protein 1 (VvXIP1) Is Regulated by Drought Stress and Transports Glycerol, Hydrogen Peroxide, Heavy Metals but Not Water
Source: PLoS One. 2016 Aug 9;11(8):e0160976. doi: 10.1371/journal.pone.0160976 (PMC4978503; doi:10.1371/journal.pone.0160976)
Supplement: S1 Table — (DOCX) [file pone.0160976.s008.docx]

**S1 Table.** Primer sequences used in this study

|  | **Primer forward (5’ – 3’)** | **Primer reverse (5’- 3’)** |
| --- | --- | --- |
| **qGAPDH** | CACGGTCAGTGGAAGCATCATGA | CCTTGTCAGTGAACACACCAGTTGACTC |
| **qVvXIP1** | ATCATGTCGGTTGTTGTTGC | CAGCGCGTGAGAAAGAGATA |
| **GATEWAY VvXIP1** | GGGGACAAGTTTGTACAAAAAA  GCAGGCTTCCAAATGGGTTCA  CACAATGGGGTTG | GGGGACCACTTTGTACAAG  AAAGCTGGGTCTATCAATG AATGCCTCAATATATTC |
